# Comparison of radiographic methods for detecting radiolucent uroliths in dogs

**DOI:** 10.1371/journal.pone.0274087

**Published:** 2022-09-22

**Authors:** Luciano Alves Faria, Adriana Érica Wilkes Burton Meirelles, Tilde Rodrigues Froes, Thassila Caccia Feragi Cintra, Daniel Peixoto Pereira, Marcela Aldrovani Rodrigues, Fernanda Nastri Gouvêa, Caio Santos Pennacchi, Najla Doutel Assaf, Leandro Zuccolotto Crivellenti

**Affiliations:** 1 Veterinary Teaching Hospital, Animal Science Graduate Program, Franca University, Franca, São Paulo, Brazil; 2 Practicing Veterinarian Radiologist in Rio Grande do Sul, Canoas, Rio Grande do Sul, Brazil; 3 Department of Veterinary Medicine, Veterinary Sciences Graduate Program, Federal University of Paraná, Curitiba, Paraná, Brazil; 4 Practicing Veterinary Clinician, Uberlândia, Minas Gerais, Brazil; 5 Graduate Program in Veterinary Science, Universidade Federal de Uberlândia, Uberlândia, Minas Gerais, Brazil; 6 Practicing Veterinary Clinician, Campos do Jordão, São Paulo, Brazil; UTAD: Universidade de Tras-os-Montes e Alto Douro, PORTUGAL

## Abstract

The purpose of this study was to evaluate and compare positive cystography techniques at 5%, 10%, and 20%, as well as three different double-contrast protocols for detecting radiolucent uroliths with a diameter of less than 3.0 mm in dogs. Six cadavers were used, one was selected at random to represent the negative control, and the others were submitted to urolith implantation in the bladder by urethral catheter. Three radiology professionals blindly accessed ventrodorsal and -lateral projections of each test. Contrast at 20% showed greater diagnostic sensitivity, but with greater difficulty identifying the number and size of the uroliths. Consequently, double-contrast techniques are better and should be used for diagnostic and therapeutic planning. Sensitivity and specificity tests demonstrated that positive 5% cystography and different concentrations of double contrast obtained better results in terms of sensitivity and specificity. However, due to the presence of a greater amount of artifacts in the 5% cystography, it is suggested that double contrast is used for this purpose, especially with the removal of contrast excess (protocol 2).

## Introduction

Urolithiasis in companion animals is a prevalent abnormality when compared to other disorders that affect the urinary tract of dogs and cats, which can cause post renal azotemia due to urinary tract obstruction. It can also be a bacterial reservoir, can lead to inflammation from tissue irritation [1, 2].

Ammonium urate uroliths represent 3–5% of the prevalence [2, 3], with the Dalmatian breed being the most affected compared to the others [3]. In felines, it has been observed more in Egyptian Mau [4]. These uroliths have a low molecular density, which makes them radiolucent on radiographic imaging. However, in this case, it is difficult to identify them with conventional radiography alone [5]. For this purpose, contrasted radiographic techniques such as positive and double-contrast cystography have been used to detect these uroliths, showing good results even when evaluating sizes smaller than 5 mm [6–8]. However, there are various forms of cystography described and no work that performs a comparison on animals.

The use of double-contrast cystography techniques proved to be superior to ultrasonography to estimate the size of a solitary cystolith [9]. Knowledge of the size and quantity of uroliths contributes greatly in the decision making for less invasive manipulation, such as the performance of urohydropropulsion techniques or retro-hydropropulsion [10, 11], or even cystoscopy to remove stones [12].

The use of different contrast concentrations can provide distinct information in the evaluation of radiolucent structures. In this sense, we sought to evaluate radiographic techniques of positive and double-contrast cystography with different concentrations of iodinated contrast to assess sensitivity and specificity in the detection of small radiolucent uroliths in dogs.

## Material and methods

### Ethical aspects

This work was approved by the Ethics Committee on the Use of Animals at the University of Franca (n. 4748070318).

### Selection of animals

The experiment was performed at the Veterinary Clinic Pronto Socorro Veterinário in Uberlândia-MG. In this study, six dogs cadavers, three females and three males were obtained from hospital care, after due release and authorization by their owners. The average weight was 7.7 ± 3.9 kg and the average age of 7.6 ± 5.2 years. Cadavers that had no abnormalities on visualization and interpretation of the bladder and urethra on x-ray were selected. As an exclusion criterion, all selected animals had previously been evaluated by survey radiograph and were free from any alteration in the anatomical conformity of the lower urinary tract, as well as overlapping artifacts like gases and feces. The animals were kept frozen until the moment of the experiment. These animals were thawed naturally in an air-conditioned environment.

### Implantation of uroliths

One of the cadavers was randomly selected as a control (no uroliths were inserted) and in the others, ammonium urate uroliths were implanted in the urinary bladder using a urethral catheter. These uroliths were obtained from clinical routine and classified according to mineral type by the Nephrology Service of the Veterinary Hospital of the University of Franca (UNIFRAN).

The selection of the number of implanted uroliths was carried out randomly, with numbers from 1 to 30 (based on our hospital routine—data not shown) being recommended for the draw. All uroliths used had sizes ranging from 1 to 3 mm, with 3, 7, 8, 12, and 15 randomly selected uroliths being inserted into the animals. The Random® application (“Aleatório”, in portuguese) version 1.2 (https://play.google.com/store/apps/details?id=com.start.devaneio.alatorio) was used to select the number of uroliths by the study supervisor, who did not perform stone counts during the examinations. In females, an episiotomy was performed to introduce the uroliths via pelvic urethra with the aid of a urethral catheter n°8—Mark Med. In males, the perineal urethra was exposed and sectioned to secure the urinary catheter n°8 –Mark Med for the introduction of uroliths, all catheters were fixed by a suture in tobacco pouch to avoid extravasation of the applied contrast. There was no accidental removal of any uroliths during contrast drainage or bladder massage.

### Radiographic projections

All animals were submitted to two radiographic projections with simple x-ray, in the lateral and ventro-dorsal positions; After this initial step, all animals were submitted to positive and double-contrast cystography radiographs (lateral and ventro-dorsal) with different contrast concentrations.

### Positive cystography

Positive cystography techniques were performed in ascending order of contrast concentration. The contrast used in all exams was Iopamidol 612 mg/ml (Iopamiron® 300, Patheon Italy S.p.a). Contrast concentrations of 5%, 10% and 20% were used, adapted from the original iodinated contrast (Omnipaque 300mg I/mL) Another two techniques (30% and 50% concentration) were performed in one animal only, due to the impossibility of diagnosing the uroliths.

The concentrations of positive cystography mentioned above were obtained from the pure contrast diluted in saline solution, and the dose of contrast applied was 10 ml/kg [6, 7]. Between each change of concentration, the bladder was emptied with the help of a 20 ml syringe coupled to the urethral catheter, the volume inserted was removed in its entirety for the beginning of the next concentration to be evaluated.

### Double-contrast cystography

Three distinct double-contrast cystography standards were performed, following established protocols. The first one (protocol 1) began with an intravesical application of 4.5 ml of pure contrast (Iopamiron® 300) per animal, followed by the application of 10 ml/kg of atmospheric air, without removing the contrast [7]. Protocol 2 started with 5 ml and 10 ml of pure contrast applied to animals weighing up to 10 kg and above 10 kg, respectively. After injecting the pure contrast, a light bladder massage was performed and then the excess contrast was removed with syringe suction. Afterward, the air was applied at a dose of 8 ml/kg. Finally, protocol 3 tested 10 ml of pure Iopamiron® 300 intravesical contrast (literature dose 5–15 ml/animal), associated with the application of 9 ml/kg of air (literature dose 6–12 ml/kg of air), without removing the contrast [7]. During the changing of contrasts of different concentrations, the urinary bladder was emptied completely, and the application of the next concentration began. At the end of the procedure, the bladder was emptied and the inserted uroliths were fully recovered.

### Radiological images and assessments

The equipment used to obtain digital images was a CDK x-ray device, model MAG VET, 320 mA and 125 kV. In total, 86 digital radiographic images were obtained. After, they were submitted to three “blind” evaluators who were veterinarians specialized in veterinary radiology with an evaluation spreadsheet.

All digital radiographic images (86 radiographic images) were obtained in DICOM [8]. Then, random codes were created containing five digits (randomly between letters and numbers)–“Random” application, version 1.2. The number of codes created was proportional to the number of radiographic images obtained, and each code was used to rename each digital image, so the images were then scrambled for later evaluation.

Access to the digital images in DICOM was available to the evaluators, which were evaluated by the number of uroliths diagnosed in the bladder (values from 0 to 30), amount of contrast (insufficient, adequate, or excessive), quality of images provided (adequate, artifacts). To be considered adequate and without artifacts, the image had to be categorized in this way by the three evaluators.

### Statistical analysis

For statistical analysis, the MedCalc® program for Windows in version 18.11.3 was used (MedCalc Software, Ostend, Belgium). Sensitivity and specificity tests were performed for each positive cystography evaluation (5%, 10%, and 20%), as well as for double-contrast protocols. As a criterion of truly positive values, 10% standard deviation was used under the total number of uroliths inserted. False-positive values were found when the number of uroliths was higher than those inserted, or if the negative control animal was categorized with the presence of uroliths. The truly negative was categorized in the negative control animal, which did not have uroliths inserted and was recognized as negative by the evaluators. Animals with the presence of uroliths, which were classified as exempt by the evaluators, these were identified as false negatives.

For the evaluation of the results among the three evaluators, the Kappa statistical method was used to assess the degree of agreement between the classes evaluated using the intraclass correlation coefficient.

This method uses a descriptive scale for values, where ρ is Pearson’s correlation coefficient, and ρ c represents the correlation coefficient, being: ρ c < 0.90 (weak agreement force); 0.90 < ρ c < 0.95 (moderate agreement strength); 0.95 < ρ c < 0.99 (substantial agreement strength); and ρ c > 0.99 (almost perfect agreement strength), and a 95% confidence interval [13–15].

## Results

The positive cystography at 20% contrast showed greater sensitivity (87.5%), but low specificity (25%). This was unlike the lower concentrations of contrast (5 and 10%) that had better accuracy (85.7% and 72.9%) and specificity (100% and 85.7%), respectively, as shown in Table 1. The double-contrast protocols 1, 2, and 3, showed similarity regarding sensitivity (75%, 71.4%, and 77.8%), specificity (75%, 80%, and 66.7%), and accuracy (75%, 75.7%, and 72.2%), respectively, with the removal of the contrast (double contrast 2) showing little superiority of visualization when compared to the other techniques (S1 Table).

**Table 1:** Sensitivity and specificity tests, accuracy, positive predictive values (PPV), negative predictive values (NPV), and intraclass correlation coefficient (ICC) in the evaluation of 86 radiographic images of dogs submitted to positive and double-contrast cystography at different contrast concentrations.

Cystography	Sensitivity	Specificity	ACU	PPV	NPV	ICC (Average value)
Contrast 5%	71.4 (29.0–96.3)	100 (47.8–100)	0.857	100	99.4	0.99 (0.97–0.99)
Contrast 10%	60 (14.7–94.7)	85.7 (42.1–99.6)	0.729	7.9	99	0.99 (0.97–0.99)
Contrast 20%	87.5 (47.3–99.7)	25 (0.6–80.6)	0.563	2.3	98.9	0.94 (0.85–0.98)
Double contrast 1	75 (34.9–96.8)	75 (19.4–96.8)	0.75	5.8	99.3	0.98 (0.95–0.99)
Double contrast 2	71.4 (29.0–96.3)	80 (28.3–99.5)	0.757	6.8	99.3	0.97 (0.92–0.99)
Double contrast 3	77.8 (40.0–97.1)	66.7 (9.4–99.2)	0.722	4.5	99.3	0.99 (0.96–0.99)

Data presented in percentage values (95% confidence interval), Accuracy (ACU), Positive Predictive Value (PPV), Negative Predictive Value (NPV), Intraclass Correlation Coefficient (ICC).

The results of the intraclass correlation coefficient demonstrated that there was a strong correlation between them in all tests (Table 1). The 20% contrast showed the lowest agreement between evaluators; however, all protocols were greater than 90%. Thirty-one (36%) of the 86 radiographic images obtained were classified as “adequate contrast” at the time of evaluation. A lack of radiographic contrast was evidenced in 13 images (15.1%), while an excess was not a consensus among the evaluators (Table 1).

Regarding image quality, 35 (40.7%) obtained the classification as exempt from possible artifacts that could interfere with the results. As for the main artifact, air bubbles were reported in the bladder or urethral tube (13; 25.5%), gases in the intestine (3; 5.9%), and bone overlap in the evaluated structures (3; 5.9%).

**Fig 1:**
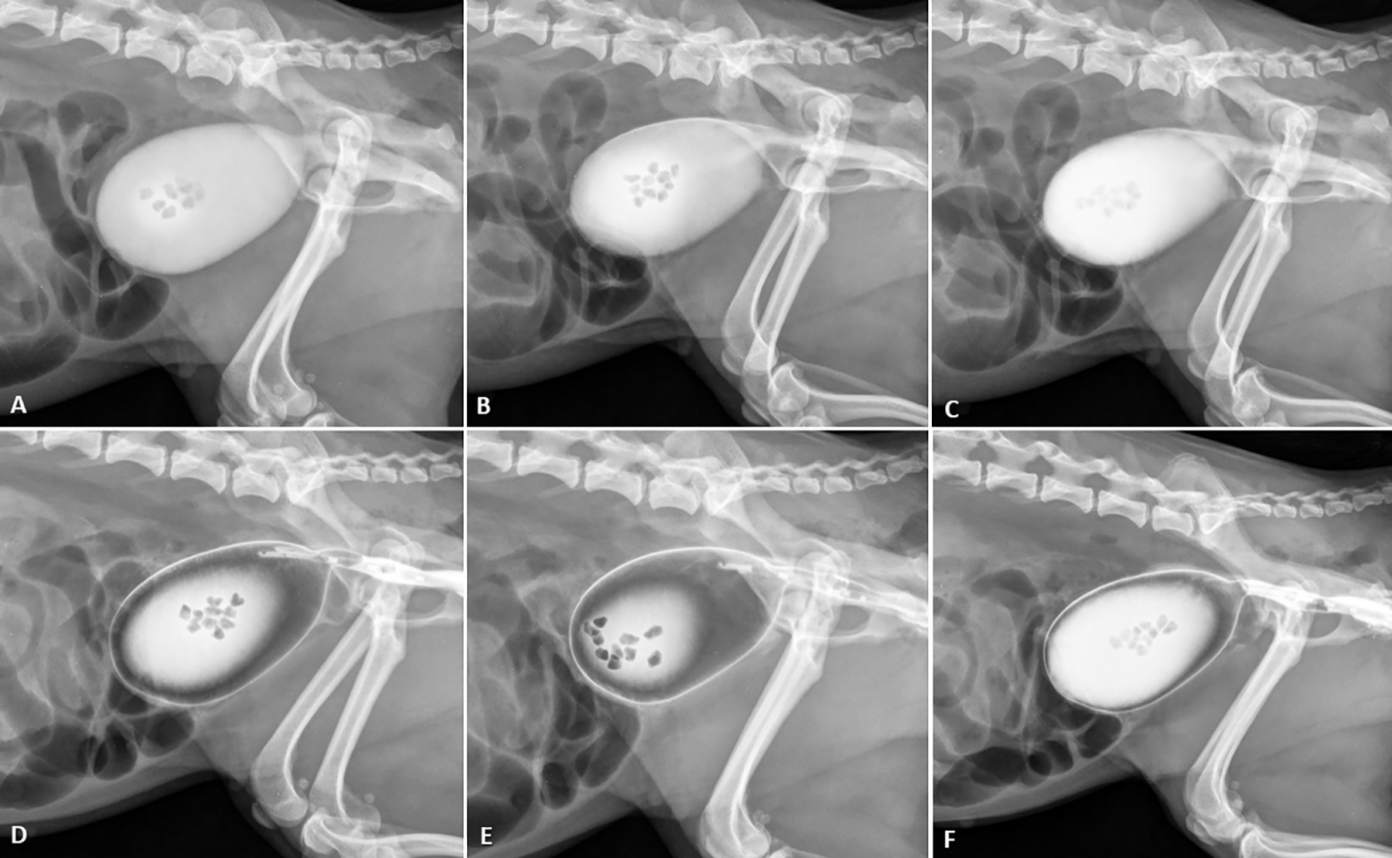
Left lateral radiographic projections using positive contrast tests and double contrast when 12 uroliths were inserted into the bladder. In (A) 5% positive contrast, showing easier visualization of uroliths; (B) 10% positive contrast; (C) 20% positive contrast; (D) double contrast 1; (E) double contrast 2; (F) double contrast 3.

The volume of iodinated contrast used and the quality of the images in positive cystography techniques (5, 10, and 20%), and the three concentrations of iodinated contrast used in double-contrast techniques are shown in Fig 1.

## Discussion

Both 5 and 20% positive cystography and double-contrast protocols had good results in the detection of uroliths and can be used in the diagnosis of radiolucent uroliths. Thus, as previously observed, the contrast at 20% showed greater diagnostic sensitivity [16]. However, this technique causes greater difficulty in identifying the number of uroliths (low specificity), showing that double-contrast tests are more suitable for treatment plans.

Although there are studies that used artificial bladders [9, 16] and Petri dishes [17, 18] to detect uroliths, the therapeutic focus was never proposed. Our study is more realistic to the medical practice, including related factors that may cause artifacts.

Double-contrast cystography allows the evaluation of the bladder wall and exceptionally detect the presence of calculi or blood clots, mainly when radiolucent structures are suspected [19, 20]. Images should be interpreted by a person highly familiar with this technique since the presence of artifacts (such as air bubbles) can misdiagnose bladder stones and/or irregularities [7].

Techniques previously described were used to minimize the presence of artifacts such as small air bubbles coming from the urethral tube inside the bladder [21]. Even with pre-filling the entire urethral tube with contrast until its insertion, 13 images were considered with the presence of an artifact for this item, with eight interfering in the precision regarding the exact number of uroliths.

As a discouraging factor for the double-contrast technique, there is a report of embolization due to pneumocystography [22]. However, due to very few cases reported in the literature, the lack of a consistent diagnosis of its correlation with the technique and our widespread use in the routine (data not provided), associated with the fact that there was no occurrence of embolization in double-contrast cystography, the technique should be used for therapeutic conduct.

Similar to double-contrast cystography, abdominal ultrasound also allows assessment of the bladder lumen and any intra-luminal structures [19]. However, this technique can overestimate the size of the calculations [9] and in the presence of a lower-frequency (5 MHz or lower) transducer, it can cause false negative results, especially with small stones [16]. These findings could not be effectively evaluated since the uroliths were similar in size.

Other advanced imaging tests, such as computed tomography (CT) and magnetic resonance, have been requested more frequently. Both allow an excellent anatomical and structural assessment of the entire urinary tract. The accuracy of CT was equivalent to double-contrast cystography, and both were superior to abdominal ultrasound [9].

Despite not being the objective of this study, the double contrast showed a better assessment of the bladder wall and may present results superior to those contrasted for the overall assessment of the bladder.

## Conclusion

A 20% positive cystography shows better results for diagnosing radiolucent uroliths smaller than 3 mm. However, the use of double-contrast tests, especially with the removal of excess contrast, allows identifying quantity and size with greater precision, being indicated to aid in therapeutic decisions.

## Supporting information

S1 Table Raw data. (DOCX)
