# Supplementary material for: Comparison of radiographic methods for detecting radiolucent uroliths in dogs
Source: PLoS One. 2022 Sep 22;17(9):e0274087. doi: 10.1371/journal.pone.0274087 (PMC9499233; doi:10.1371/journal.pone.0274087)
Supplement: S1 Table — (DOCX) [file pone.0274087.s001.docx]

**Supporting Information files**

| **Animal code** | **Uroliths - Bladder** | | | **Uroliths - Urethra** | | | **Total** | | | **Technique** | **True amount of implanted uroliths** |
| --- | --- | --- | --- | --- | --- | --- | --- | --- | --- | --- | --- |
|  | **BE1** | **BE2** | **BE3** | **BE1** | **BE2** | **BE3** | **BE1** | **BE2** | **BE3** |  |  |
| LC5ZZ | 0 | 0 | 0 | 0 | 0 | 0 | 0 | 0 | 0 | Simple  X-Ray | 0 |
| 45MU5 | 0 | 0 | 0 | 0 | 0 | 0 | 0 | 0 | 0 |  |  |
| 3HMWS | 0 | 0 | 0 | 0 | 0 | 0 | 0 | 0 | 0 |  |  |
| 9DPCB | 0 | 0 | 0 | 0 | 0 | 0 | 0 | 0 | 0 |  |  |
| 3VMI0 | 0 | 0 | 1 | 0 | 0 | 0 | 0 | 0 | 1 | Contrast 5% |  |
| 6GF0G | 0 | 0 | 0 | 0 | 0 | 0 | 0 | 0 | 0 |  |  |
| YWSWQ | 0 | 0 | 1 | 0 | 0 | 0 | 0 | 0 | 1 | Contrast 10% |  |
| J013J | 0 | 0 | 0 | 0 | 0 | 0 | 0 | 0 | 0 |  |  |
| FK3KU | 0 | 0 | 2 | 0 | 0 | 0 | 0 | 0 | 2 | Contrast 20% |  |
| 8U0C5 | 1 | 0 | 0 | 0 | 0 | 0 | 1 | 0 | 0 |  |  |
| YRS6L | 0 | 0 | 0 | 0 | 0 | 0 | 0 | 0 | 0 | Double contrast 1 |  |
| 0YRLU | 0 | 0 | 0 | 0 | 0 | 0 | 0 | 0 | 0 |  |  |
| WQG1D | 0 | 0 | 0 | 0 | 0 | 0 | 0 | 0 | 0 | Double contrast 2 |  |
| E0TCL | 0 | 0 | 0 | 0 | 0 | 0 | 0 | 0 | 0 |  |  |
| XSUMC | 0 | 0 | 0 | 0 | 0 | 0 | 0 | 0 | 0 | Double contrast 3 |  |
| K7LDA | 0 | 0 | 0 | 0 | 0 | 0 | 0 | 0 | 0 |  |  |
| VKI1J | 0 | 0 | 3 | 0 | 0 | 0 | 0 | 0 | 3 | Simple  X-Ray | 3 |
| QI1YG | 0 | 0 | 0 | 0 | 0 | 0 | 0 | 0 | 0 |  |  |
| FFM4F | 3 | 3 | 3 | 0 | 0 | 0 | 3 | 3 | 3 | Contrast 5% |  |
| 88MI3 | 0 | 0 | 0 | 0 | 0 | 0 | 0 | 0 | 0 |  |  |
| 2CF7D | 2 | 3 | 6 | 0 | 0 | 0 | 2 | 3 | 6 | Contrast 10% |  |
| 3095V | 0 | 0 | 0 | 0 | 0 | 0 | 0 | 0 | 0 |  |  |
| 36SRS | 3 | 3 | 3 | 0 | 0 | 0 | 3 | 3 | 3 | Contrast 20% |  |
| J3B4M | 3 | 3 | 3 | 1 | 0 | 1 | 4 | 3 | 4 |  |  |
| OQERT | 3 | 2 | 2 | 0 | 0 | 0 | 3 | 2 | 2 | Double contrast 1 |  |
| 0HUWL | 3 | 3 | 3 | 0 | 0 | 0 | 3 | 3 | 3 |  |  |
| ZP6Q7 | 3 | 3 | 3 | 0 | 0 | 1 | 3 | 3 | 4 | Double contrast 2 |  |
| 9MQE0 | 3 | 3 | 3 | 0 | 0 | 1 | 3 | 3 | 4 |  |  |
| G87F2 | 3 | 3 | 5 | 0 | 0 | 0 | 3 | 3 | 5 | Double contrast 3 |  |
| WHQSA | 3 | 3 | 3 | 0 | 0 | 0 | 3 | 3 | 3 |  |  |
| LF5CT | 0 | 2 | 2 | 0 | 0 | 0 | 0 | 2 | 2 | Simple  X-Ray | 7 |
| 8M41V | 0 | 3 | 0 | 0 | 0 | 0 | 0 | 3 | 0 |  |  |
| 3G06L | 3 | 2 | 4 | 4 | 4 | 5 | 7 | 6 | 9 | Contrast 5% |  |
| HAPRU | 2 | 2 | 2 | 4 | 4 | 2 | 6 | 6 | 4 |  |  |
| BV7ZQ | 4 | 4 | 4 | 0 | 0 | 0 | 4 | 4 | 4 | Contrast 10% |  |
| 5BWGU | 0 | 0 | 0 | 0 | 0 | 0 | 0 | 0 | 0 |  |  |
| 31QA6 | 4 | 4 | 4 | 3 | 3 | 3 | 7 | 7 | 7 | Contrast 20% |  |
| CEBWU | 3 | 3 | 3 | 3 | 3 | 3 | 6 | 6 | 6 |  |  |
| KKCET | 4 | 4 | 4 | 3 | 2 | 2 | 7 | 6 | 6 | Double contrast 1 |  |
| 9X174 | 4 | 4 | 9 | 4 | 3 | 3 | 8 | 7 | 12 |  |  |
| WQ98V | 4 | 4 | 5 | 3 | 0 | 1 | 7 | 4 | 6 | Double contrast 2 |  |
| 9LALZ | 4 | 4 | 4 | 3 | 3 | 5 | 7 | 7 | 9 |  |  |
| WUR63 | 4 | 4 | 4 | 3 | 3 | 3 | 7 | 7 | 7 | Double contrast 3 |  |
| 8WN6H | 4 | 4 | 4 | 2 | 2 | 0 | 6 | 6 | 4 |  |  |
| UHIDJ | 4 | 4 | 6 | 0 | 0 | 0 | 4 | 4 | 6 | Simple  X-Ray | 8 |
| HWUIT | 0 | 0 | 0 | 0 | 0 | 0 | 0 | 0 | 0 |  |  |
| R8015 | 9 | 8 | 9 | 0 | 0 | 0 | 9 | 8 | 9 | Contrast 5% |  |
| L5U6W | 6 | 7 | 5 | 0 | 0 | 0 | 6 | 7 | 6 |  |  |
| RQ10H | 9 | 8 | 10 | 0 | 0 | 0 | 9 | 8 | 9 | Contrast 10% |  |
| GGTJ2 | 8 | 8 | 10 | 0 | 0 | 0 | 8 | 8 | 8 |  |  |
| CZ4GU | 9 | 9 | 9 | 1 | 0 | 0 | 10 | 9 | 9 | Contrast 20% |  |
| A4892 | 8 | 8 | 11 | 0 | 0 | 0 | 8 | 8 | 8 |  |  |
| JS300 | 2 | 2 | 2 | 0 | 0 | 0 | 2 | 2 | 2 | Double contrast 1 |  |
| TXGCF | 6 | 6 | 5 | 0 | 0 | 1 | 6 | 6 | 6 |  |  |
| O0RVH | 1 | 1 | 1 | 0 | 0 | 0 | 1 | 1 | 1 | Double contrast 2 |  |
| ZHD0B | 9 | 8 | 8 | 5 | 0 | 2 | 14 | 8 | 9 |  |  |
| LPCGJ | 4 | 4 | 4 | 0 | 0 | 2 | 4 | 4 | 4 | Double contrast 3 |  |
| I3W02 | 8 | 8 | 8 | 1 | 0 | 0 | 9 | 8 | 8 |  |  |
| Q5VS3 | 2 | 5 | 8 | 0 | 0 | 0 | 2 | 5 | 8 | Simple  X-Ray | 12 |
| BGKN8 | 0 | 0 | 0 | 0 | 0 | 0 | 0 | 0 | 0 |  |  |
| M7215 | 7 | 8 | 9 | 0 | 0 | 0 | 7 | 8 | 9 | Contrast 5% |  |
| BTSA6 | 1 | 0 | 2 | 0 | 0 | 0 | 1 | 0 | 2 |  |  |
| EI61B | 10 | 10 | 10 | 0 | 0 | 1 | 10 | 10 | 11 | Contrast 10% |  |
| PRC7C | 10 | 10 | 10 | 1 | 1 | 1 | 11 | 11 | 11 |  |  |
| 02AMC | 10 | 10 | 17 | 1 | 3 | 5 | 11 | 13 | 22 | Contrast 20% |  |
| 03VD6 | 10 | 10 | 11 | 2 | 2 | 0 | 12 | 12 | 11 |  |  |
| 9KYB2 | 12 | 10 | 13 | 1 | 1 | 0 | 13 | 11 | 13 | Double contrast 1 |  |
| BCHW3 | 10 | 10 | 11 | 1 | 1 | 1 | 11 | 11 | 12 |  |  |
| JONBP | 10 | 10 | 10 | 1 | 0 | 2 | 11 | 10 | 12 | Double contrast 2 |  |
| QHW5O | 8 | 8 | 8 | 0 | 0 | 1 | 8 | 8 | 9 |  |  |
| S0TXM | 11 | 10 | 12 | 1 | 0 | 1 | 12 | 10 | 13 | Double contrast 3 |  |
| 3NDV7 | 10 | 10 | 10 | 2 | 1 | 1 | 12 | 11 | 11 |  |  |
| 21H1L | 15 | 13 | 18 | 0 | 0 | 0 | 15 | 13 | 18 | Simple  X-Ray | 15 |
| 1Y622 | 15 | 15 | 15 | 0 | 0 | 0 | 15 | 15 | 15 |  |  |
| KOA25 | 11 | 11 | 15 | 0 | 0 | 0 | 11 | 11 | 15 | Contrast 5% |  |
| V89WM | 10 | 10 | 13 | 0 | 0 | 0 | 10 | 10 | 13 |  |  |
| Y7709 | 14 | 15 | 17 | 0 | 0 | 0 | 14 | 15 | 17 | Contrast 10% |  |
| F6QGQ | 7 | 7 | 15 | 0 | 0 | 0 | 7 | 7 | 15 |  |  |
| MICYM | 2 | 3 | 3 | 0 | 0 | 0 | 2 | 3 | 3 | Contrast 20% |  |
| 1MLYQ | 0 | 0 | 0 | 0 | 0 | 0 | 0 | 0 | 0 |  |  |
| HFTJD | 14 | 15 | 20 | 0 | 0 | 0 | 14 | 15 | 20 | Double contrast 1 |  |
| I4FFF | 13 | 16 | 17 | 0 | 0 | 0 | 13 | 16 | 17 |  |  |
| G0JGV | 9 | 10 | 10 | 0 | 0 | 0 | 9 | 10 | 10 | Double contrast 2 |  |
| J4ZGS | 10 | 9 | 15 | 0 | 0 | 0 | 10 | 9 | 15 |  |  |
| OZC8O | 14 | 14 | 15 | 0 | 0 | 0 | 14 | 14 | 15 | Double contrast 3 |  |
| FE7FN | 13 | 12 | 15 | 0 | 0 | 1 | 13 | 12 | 16 |  |  |

BE1: blind evaluator #1; BE2: blind evaluator #2; BE3: blind evaluator #3.

|  | **Animal code** | **BE1** | **BE2** | **BE3** | **Average** | **Variation** | | **True amount of implanted uroliths** |
| --- | --- | --- | --- | --- | --- | --- | --- | --- |
| **5% contrast** | KOA25 | 11 | 11 | 15 | 12 | 14 | 17 | 15 |
|  | V89WM | 10 | 10 | 13 | 11 | 14 | 17 | 15 |
|  | 3VMI0 | 0 | 0 | 1 | 0 | 0 | 0 | 0 |
|  | 6GF0G | 0 | 0 | 0 | 0 | 0 | 0 | 0 |
|  | M7215 | 7 | 8 | 9 | 8 | 11 | 13 | 12 |
|  | BTSA6 | 1 | 0 | 2 | 1 | 11 | 13 | 12 |
|  | FFM4F | 3 | 3 | 3 | 3 | 3 | 3 | 3 |
|  | 88MI3 | 0 | 0 | 0 | 0 | 3 | 3 | 3 |
|  | R8015 | 9 | 8 | 9 | 9 | 7 | 9 | 8 |
|  | L5U6W | 6 | 7 | 5 | 6 | 7 | 9 | 8 |
|  | 3G06L | 7 | 6 | 9 | 7 | 6 | 8 | 7 |
|  | HAPRU | 6 | 6 | 4 | 5 | 6 | 8 | 7 |

**DIAGNOSTIC TEST**

**Results**

| Sensitivity | 71,429% | 29,042% to 96,331% |
| --- | --- | --- |
| Specificity | 100,000% | 47,818% to 100,000% |
| AUC | 0,857 | 0,543 to 0,986 |
| Positive Likelihood Ratio |  |  |
| Negative Likelihood Ratio | 0,286 | 0,089 to 0,922 |
| Prevalence | 2,000% |  |
| Positive Predictive Value | 100,000% |  |
| Negative Predictive Value | 99,420% | 98,154% to 99,820% |

**Intraclass correlation coefficient**

| Number of subjects (n) | 12 |
| --- | --- |
| Number of raters (k) | 3 |
| Model | The same rater for all subjects. Two-way model |
| Type | Consistency |
| Measurements | BE1, BE2, BE3 |

**Intraclass correlation coefficient**

|  | Intraclass correlation ^a^ | 95% confidence interval |
| --- | --- | --- |
| Single measures ^b^ | 0,9636 | 0,9073 to 0,9884 |
| Average measures ^c^ | 0,9876 | 0,9671 to 0,9961 |

^a^ The degree of consistency among measurements.

^b^ Estimates the reliability of single ratings.

^c^ Estimates the reliability of averages of *k* ratings.

|  | **Animal code** | **BE1** | **BE2** | **BE3** | **Average** | **Variation** | | **True amount of implanted uroliths** |
| --- | --- | --- | --- | --- | --- | --- | --- | --- |
| **10% contrast** | Y7709 | 14 | 15 | 17 | 15 | 14 | 17 | 15 |
|  | F6QGQ | 7 | 7 | 15 | 10 | 14 | 17 | 15 |
|  | YWSWQ | 0 | 0 | 1 | 0 | 0 | 0 | 0 |
|  | J013J | 0 | 0 | 0 | 0 | 0 | 0 | 0 |
|  | EI61B | 10 | 10 | 11 | 10 | 11 | 13 | 12 |
|  | PRC7C | 11 | 11 | 11 | 11 | 11 | 13 | 12 |
|  | 2CF7D | 2 | 3 | 6 | 4 | 3 | 3 | 3 |
|  | 3095V | 0 | 0 | 0 | 0 | 3 | 3 | 3 |
|  | RQ10H | 9 | 8 | 10 | 9 | 7 | 9 | 8 |
|  | GGTJ2 | 8 | 8 | 10 | 9 | 7 | 9 | 8 |
|  | BV7ZQ | 4 | 4 | 4 | 4 | 6 | 8 | 7 |
|  | 5BWGU | 0 | 0 | 0 | 0 | 6 | 8 | 7 |

**DIAGNOSTIC TEST**

**Results**

| Sensitivity | 60,000% | 14,729% to 94,702% |
| --- | --- | --- |
| Specificity | 85,749% | 42,189 to 99,662% |
| AUC | 0,729 | 0,307 to 0,897 |
| Positive Likelihood Ratio |  |  |
| Negative Likelihood Ratio |  |  |
| Prevalence | 2,000% |  |
| Positive Predictive Value | 7,912% |  |
| Negative Predictive Value | 98,997% |  |

**Intraclass correlation coefficient**

| Number of subjects (n) | 12 |
| --- | --- |
| Number of raters (k) | 3 |
| Model | The same rater for all subjects. Two-way model |
| Type | Consistency |
| Measurements | BE1, BE2, BE3 |

**Intraclass correlation coefficient**

|  | Intraclass correlation ^a^ | 95% confidence interval |
| --- | --- | --- |
| Single measures ^b^ | 0,9694 | 0,9218 to 0,9903 |
| Average measures ^c^ | 0,9896 | 0,9725 to 0,9967 |

^a^ The degree of consistency among measurements.

^b^ Estimates the reliability of single ratings.

^c^ Estimates the reliability of averages of *k* ratings.

|  | **Animal code** | **BE1** | **BE2** | **BE3** | **Average** | **Variation** | | **True amount of implanted uroliths** |
| --- | --- | --- | --- | --- | --- | --- | --- | --- |
| **20% contrast** | MICYM | 2 | 3 | 3 | 3 | 14 | 17 | 15 |
|  | 1MLYQ | 0 | 0 | 0 | 0 | 14 | 17 | 15 |
|  | FK3KU | 0 | 0 | 2 | 1 | 0 | 0 | 0 |
|  | 8U0C5 | 1 | 0 | 0 | 0 | 0 | 0 | 0 |
|  | 02AMC | 11 | 13 | 22 | 15 | 11 | 13 | 12 |
|  | 03VD6 | 12 | 12 | 11 | 12 | 11 | 13 | 12 |
|  | 36SRS | 3 | 3 | 3 | 3 | 3 | 3 | 3 |
|  | J3B4M | 4 | 3 | 4 | 4 | 3 | 3 | 3 |
|  | CZ4GU | 10 | 9 | 9 | 9 | 7 | 9 | 8 |
|  | A4892 | 8 | 8 | 11 | 9 | 7 | 9 | 8 |
|  | 31QA6 | 7 | 7 | 7 | 7 | 6 | 8 | 7 |
|  | CEBWU | 6 | 6 | 6 | 6 | 6 | 8 | 7 |

**DIAGNOSTIC TEST**

**Results**

| Sensitivity | 87,500% | 47,349% to 99,684% |
| --- | --- | --- |
| Specificity | 25,000% | 0,631% to 80,588% |
| AUC | 0,563 | 0,260 to 0,834 |
| Positive Likelihood Ratio | 1,167 | 0,625 to 2,176 |
| Negative Likelihood Ratio | 0,500 | 0,041 to 6,082 |
| Prevalence | 2,000% |  |
| Positive Predictive Value | 2,326% | 1,260% to 4,253% |
| Negative Predictive Value | 98,990% | 88,958% to 99,916% |

**Intraclass correlation coefficient**

| Number of subjects (n) | 12 |
| --- | --- |
| Number of raters (k) | 3 |
| Model | The same rater for all subjects. Two-way model |
| Type | Consistency |
| Measurements | BE1, BE2, BE3 |

**Intraclass correlation coefficient**

|  | Intraclass correlation ^a^ | 95% confidence interval |
| --- | --- | --- |
| Single measures ^b^ | 0,8499 | 0,6589 to 0,9496 |
| Average measures ^c^ | 0,9444 | 0,8529 to 0,9826 |

^a^ The degree of consistency among measurements.

^b^ Estimates the reliability of single ratings.

^c^ Estimates the reliability of averages of *k* ratings.

|  | **Animal code** | **BE1** | **BE2** | **BE3** | **Average** | **Variation** | | **True amount of implanted uroliths** |
| --- | --- | --- | --- | --- | --- | --- | --- | --- |
| **Double Contrast - Protocol 1** | HFTJD | 14 | 15 | 20 | 16 | 0 | 17 | 15 |
|  | I4FFF | 13 | 16 | 17 | 15 | 14 | 17 | 15 |
|  | YRS6L | 0 | 0 | 0 | 0 | 0 | 0 | 0 |
|  | 0YRLU | 0 | 0 | 0 | 0 | 0 | 0 | 0 |
|  | 9KYB2 | 13 | 11 | 13 | 12 | 11 | 13 | 12 |
|  | BCHW3 | 11 | 11 | 12 | 11 | 11 | 13 | 12 |
|  | OQERT | 3 | 2 | 2 | 2 | 3 | 3 | 3 |
|  | 0HUWL | 3 | 3 | 3 | 3 | 3 | 3 | 3 |
|  | JS300 | 2 | 2 | 2 | 2 | 7 | 9 | 8 |
|  | TXGCF | 6 | 6 | 6 | 6 | 7 | 9 | 8 |
|  | KKCET | 7 | 6 | 6 | 6 | 6 | 8 | 7 |
|  | 9X174 | 8 | 7 | 12 | 9 | 6 | 8 | 7 |

**DIAGNOSTIC TEST**

**Results**

| Sensitivity | 75,000% | 34,914% to 96,815% |
| --- | --- | --- |
| Specificity | 75,009% | 19,441% to 96,793% |
| AUC | 0,750 | 0,564 to 0,891 |
| Positive Likelihood Ratio |  |  |
| Negative Likelihood Ratio | 0,250 |  |
| Prevalence | 2,000% |  |
| Positive Predictive Value | 5,809% |  |
| Negative Predictive Value | 99,327% |  |

**Intraclass correlation coefficient**

| Number of subjects (n) | 12 |
| --- | --- |
| Number of raters (k) | 3 |
| Model | The same rater for all subjects. Two-way model |
| Type | Consistency |
| Measurements | BE1, BE2, BE3 |

**Intraclass correlation coefficient**

|  | Intraclass correlation ^a^ | 95% confidence interval |
| --- | --- | --- |
| Single measures ^b^ | 0,9521 | 0,8597 to 0,9879 |
| Average measures ^c^ | 0,9835 | 0,9484 to 0,9959 |

^a^ The degree of consistency among measurements.

^b^ Estimates the reliability of single ratings.

^c^ Estimates the reliability of averages of *k* ratings.

|  | **Animal code** | **BE1** | **BE2** | **BE3** | **Average** | **Variation** | | **True amount of implanted uroliths** |
| --- | --- | --- | --- | --- | --- | --- | --- | --- |
| **Double Contrast - Protocol 2** | G0JGV | 9 | 10 | 10 | 10 | 14 | 17 | 15 |
|  | J4ZGS | 10 | 9 | 15 | 11 | 14 | 17 | 15 |
|  | WQG1D | 0 | 0 | 0 | 0 | 0 | 0 | 0 |
|  | E0TCL | 0 | 0 | 0 | 0 | 0 | 0 | 0 |
|  | JONBP | 11 | 10 | 12 | 11 | 11 | 13 | 12 |
|  | QHW5O | 8 | 8 | 9 | 8 | 11 | 13 | 12 |
|  | ZP6Q7 | 3 | 3 | 4 | 3 | 3 | 3 | 3 |
|  | 9MQE0 | 3 | 3 | 4 | 3 | 3 | 3 | 3 |
|  | O0RVH | 1 | 1 | 1 | 1 | 7 | 9 | 8 |
|  | ZHD0B | 14 | 8 | 10 | 11 | 7 | 9 | 8 |
|  | WQ98V | 7 | 4 | 6 | 6 | 6 | 8 | 7 |
|  | 9LALZ | 7 | 7 | 9 | 8 | 6 | 8 | 7 |

**DIAGNOSTIC TEST**

**Results**

| Sensitivity | 71,400% | 29,098% to 96,299% |
| --- | --- | --- |
| Specificity | 80,000% | 28,307% to 99,492% |
| AUC | 0,757 |  |
| Positive Likelihood Ratio |  |  |
| Negative Likelihood Ratio |  |  |
| Prevalence | 2,000% |  |
| Positive Predictive Value | 6,812% |  |
| Negative Predictive Value | 99,289% |  |

**Intraclass correlation coefficient**

| Number of subjects (n) | 12 |
| --- | --- |
| Number of raters (k) | 3 |
| Model | The same rater for all subjects. Two-way model |
| Type | Consistency |
| Measurements | BE1, BE2, BE3 |

**Intraclass correlation coefficient**

|  | Intraclass correlation ^a^ | 95% confidence interval |
| --- | --- | --- |
| Single measures ^b^ | 0,9115 | 0,7865 to 0,9711 |
| Average measures ^c^ | 0,9686 | 0,9170 to 0,9902 |

^a^ The degree of consistency among measurements.

^b^ Estimates the reliability of single ratings.

^c^ Estimates the reliability of averages of *k* ratings.

|  | **Animal code** | **BE1** | **BE2** | **BE3** | **Average** | **Variation** | | **True amount of implanted uroliths** |
| --- | --- | --- | --- | --- | --- | --- | --- | --- |
| **Double Contrast - Protocol 3** | OZC8O | 14 | 14 | 15 | 14 | 14 | 17 | 15 |
|  | FE7FN | 13 | 12 | 16 | 14 | 14 | 17 | 15 |
|  | XSUMC | 0 | 0 | 0 | 0 | 0 | 0 | 0 |
|  | K7LDA | 0 | 0 | 0 | 0 | 0 | 0 | 0 |
|  | S0TXM | 12 | 10 | 13 | 12 | 11 | 13 | 12 |
|  | 3NDV7 | 12 | 11 | 11 | 11 | 11 | 13 | 12 |
|  | G87F2 | 3 | 3 | 5 | 4 | 3 | 3 | 3 |
|  | WHQSA | 3 | 3 | 3 | 3 | 3 | 3 | 3 |
|  | LPCGJ | 4 | 4 | 6 | 5 | 7 | 9 | 8 |
|  | I3W02 | 9 | 8 | 8 | 8 | 7 | 9 | 8 |
|  | WUR63 | 7 | 7 | 7 | 7 | 6 | 8 | 7 |
|  | 8WN6H | 6 | 6 | 4 | 5 | 6 | 8 | 7 |

**DIAGNOSTIC TEST**

**Results**

| Sensitivity | 77,778% | 40,019% to 97,186% |
| --- | --- | --- |
| Specificity | 66,667% | 9,430% to 99,160% |
| AUC | 0,722 | 0,401 to 0,931 |
| Positive Likelihood Ratio | 2,333 | 0,454 to 12,005 |
| Negative Likelihood Ratio | 0,333 | 0,077 to 1,437 |
| Prevalence | 2,000% |  |
| Positive Predictive Value | 4,545% | 0,917% to 19,678% |
| Negative Predictive Value | 99,324% | 97,152% to 99,842% |

**Intraclass correlation coefficient**

| Number of subjects (n) | 12 |
| --- | --- |
| Number of raters (k) | 3 |
| Model | The same rater for all subjects. Two-way model |
| Type | Consistency |
| Measurements | BE1, BE2, BE3 |

**Intraclass correlation coefficient**

|  | Intraclass correlation ^a^ | 95% confidence interval |
| --- | --- | --- |
| Single measures ^b^ | 0,9658 | 0,9127 to 0,9891 |
| Average measures ^c^ | 0,9883 | 0,9691 to 0,9964 |

^a^ The degree of consistency among measurements.

^b^ Estimates the reliability of single ratings.

^c^ Estimates the reliability of averages of *k* ratings.
